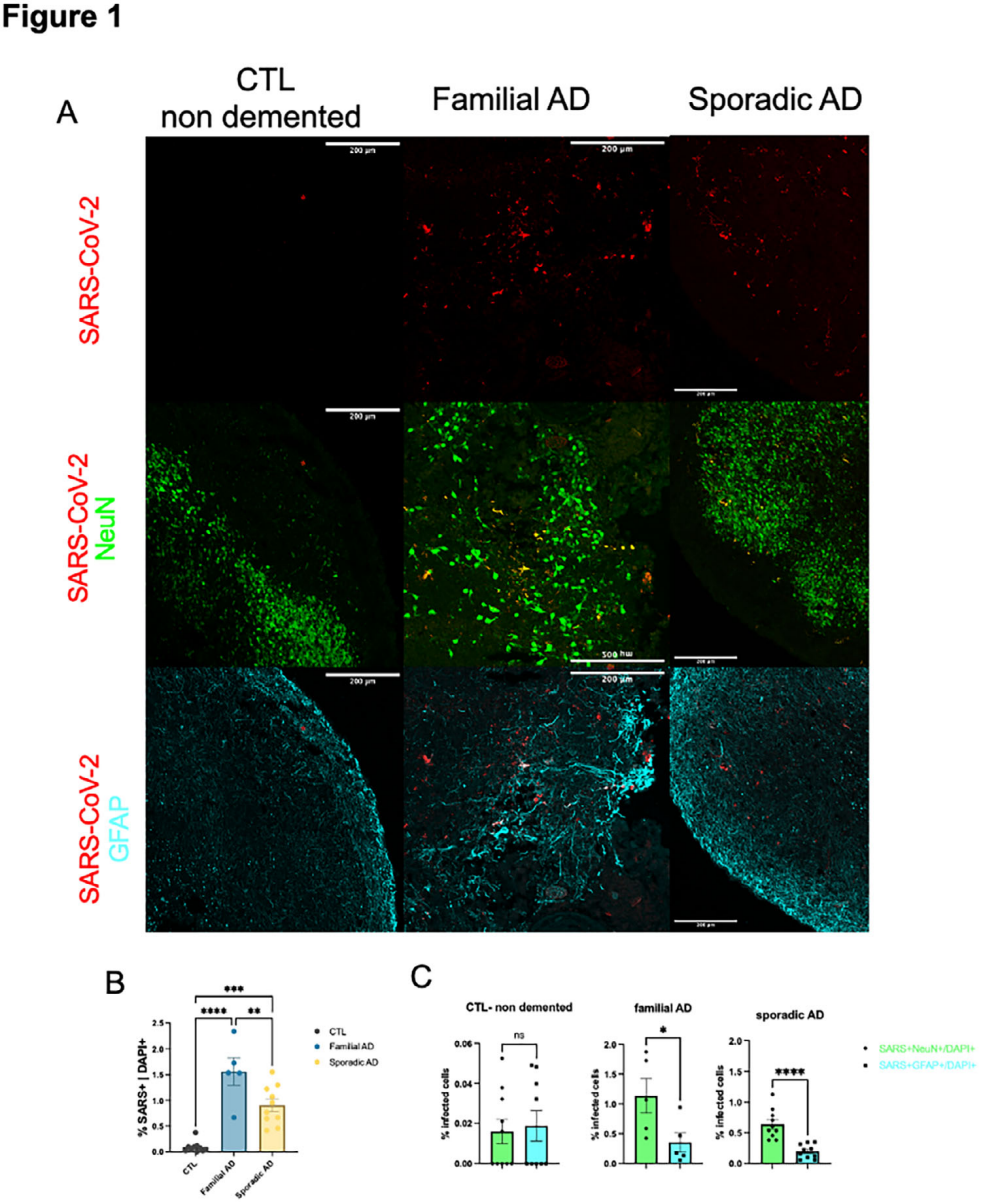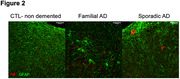# Unravelling neurodegenerative effects of SARS‐CoV‐2 infection in Alzheimer’s disease‐ brain organoids

**DOI:** 10.1002/alz.092158

**Published:** 2025-01-03

**Authors:** Laura Garcia Gonzalez, Andrea Martí Sarrias, Maria Carmen Puertas, Isabel Turpin, Ángel Bayón Gil, Patricia Resa Infante, Javier Martinez Picado, Arcadi Navarro, Sandra Acosta

**Affiliations:** ^1^ Barcelonaβeta Brain Research Center (BBRC), Pasqual Maragall Foundation, Barcelona Spain; ^2^ Institute of Neurosciences, L'Hospitalet de Llobregat, Barcelona Spain; ^3^ Institute of Evolutionary Biology (UPF‐CSIC), Barcelona Spain; ^4^ IrsiCaixa AIDS Research Institute, Badalona, Barcelona Spain; ^5^ CIBERINFEC, Instituto de Salud Carlos III, Madrid Spain; ^6^ Germans Trias i Pujol Research Institute (IGTP), Badalona, Barcelona Spain; ^7^ University of Vic‐Central University of Catalonia, Vic, Barcelona Spain; ^8^ Catalan Institution for Research and Advanced Studies, Barcelona Spain; ^9^ Centre for Genomic Regulation (CRG), Barcelona Institute of Science and Technology (BIST), Barcelona Spain; ^10^ Institute for Biomedical Research of Bellvitge, L'Hospitalet de Llobregat, Barcelona Spain

## Abstract

**Background:**

The increased vulnerability of Alzheimer’s disease patients to severe SARS‐CoV‐2 infection raises crucial concerns, especially with the potential transition of the COVID‐19 pandemic to an endemic state. Given the rising prevalence of Alzheimer’s in an aging world‐wide population, elucidating whether SARS‐CoV‐2 infection may induce or accelerate neurodegeneration becomes imperative.

**Method:**

To investigate the neurodegenerative effects of SARS‐CoV‐2 infection, we generated brain organoids using human induced pluripotent stem lines from one non‐demented control, one with sporadic Alzheimer’s, and one with familial Alzheimer’s. After 6 months of differentiation, aged organoids were infected with SARS‐CoV‐2 in duplicates (n = 4 organoids/each). Post‐infected organoids were analyzed systematically 6 weeks post‐infection in order to simulate long‐lasting viral effects. We collected cell media, and fixed the brain organoids to monitor cellular infection, the status of neurons, the reactivity of astrocytic glia, and the presence of neurodegeneration markers.

**Result:**

Sustained low‐level virus replication was detected in the organoids medium for 6 weeks. We observed a higher proportion of infected cells in Alzheimer’s‐derived organoids compared to the control organoids (Fig. 1A and Fig. 1B). In addition, neuronal infection rates surpassed astrocytes across all genotypes (Fig. 1C). RNA expression analysis of SARS‐CoV‐2 receptors expression showed no significant differences between different genotypes, suggesting that the differences in infection rates is not solely attributable to receptor expression. We found no significant differences in the total number of neurons or astrocytes, nor the proportion of caspase‐3 positive cells. However, an increase in β‐amyloid peptide counts and particle area along with an elevated Aβ42/ Aβ40 ratio was observed after infection (Fig. 2). Regarding Tau phosphorylation levels, there were no clear differences between genotypes or conditions.

**Conclusion:**

Our research reveals a heightened susceptibility of Alzheimer’s disease‐derived brain organoids to SARS‐CoV‐2 infection together with a notable accumulation of Aβ peptides. These findings suggest a potential link between viral exposure and exacerbation of neurodegenerative markers in Alzheimer’s disease, underscoring the urgent need for further exploration of this relationship amid the evolving landscape of the COVID‐19 pandemic.